# Deficiency of 14-3-3ε and 14-3-3ζ by the *Wnt1* promoter-driven Cre recombinase results in pigmentation defects

**DOI:** 10.1186/s13104-016-1980-z

**Published:** 2016-03-22

**Authors:** Brett Cornell, Kazuhito Toyo-oka

**Affiliations:** Department of Neurobiology and Anatomy, Drexel University College of Medicine, Philadelphia, PA 19129 USA

**Keywords:** 14-3-3, *Ywhae*, *Ywhaz*, Knockout mouse, Cre transgenic mouse, Neural crest cell, Pigmentation, White patch, Melanocyte, Weight modulation

## Abstract

**Background:**

The seven 14-3-3 protein isoforms bind to numerous proteins and are involved in a wide variety of cellular events, including the cell cycle, cell division, apoptosis and cancer. We previously found the importance of 14-3-3 proteins in neuronal migration of pyramidal neurons in the developing cortex. Here, we test the function of 14-3-3 proteins in the development of neural crest cells in vivo using mouse genetic approaches.

**Results:**

We found that 14-3-3 proteins are important for the development of neural crest cells, in particular for the pigmentation of the fur on the ventral region of mice.

**Conclusions:**

Our data obtained from the *14*-*3*-*3ε/14*-*3*-*3ζ/Wnt1*-*Cre* mice strongly indicate the importance of 14-3-3 proteins in the development of melanocyte lineages.

## Background

The 14-3-3 protein family is composed of seven isoforms, which are encoded by separate genes [1]. By producing and analyzing *14-3-3ε* conditional knockout mice and *14-3-3ζ* conventional knockout mice, we found that these 14-3-3 proteins are important for neurogenesis of neuronal progenitor cells and neuronal migration of pyramidal neurons in the developing cortex [2, 3]. Also, these knockout mice showed several behavioral defects, such as learning and memory defects and seizures [2, 4, 5]. In addition to the importance of 14-3-3 proteins in neural development and neurological diseases, 14-3-3*ε* is important for proper heart development [6]. Taken together with the fact that 14-3-3 proteins are important for other cellular events including cancer development and metabolism [7–10], it is evident that 14-3-3 proteins are important for multiple cellular events which are essential for the correct development and function of a variety of tissues.

The repeated-epilation (Er) mutant mouse, as first reported by Hunsicker in 1960, is characterized by a loss of hair induced by radiation exposure [11]. Homozygote Er mice die at birth while heterozygotes develop normally, followed by excessive hair loss resulting in a sparse coat. Li et al. [12] showed that the repeated epilation is caused by a single nucleotide insertion in the *Sfn* gene, encoding the 14-3-3σ protein. In addition, they found that the skin defects seen in these mice are the result of abnormal epidermal differentiation. Thus, this indicates that 14-3-3σ proteins are important for the proper development of the epidermis.

Miller-Dieker syndrome is characterized by severe lissencephaly caused by neuronal migration defects as well as craniofacial defects [13] and is caused by a chromosomal deletion in the 17p13.3 region where the *Lis1 (PAFAH1B1)* and *14*-*3*-*3ε (YWHAE)* genes are localized. The *14*-*3*-*3ε* and *Lis1* knockout mice do not show any craniofacial defects, suggesting that the 14-3-3*ε* protein is not important for craniofacial development. In general, 14-3-3 proteins have to form homodimers or heterodimers to function inside cells, depending on each 14-3-3 isoform. Although 14-3-3ε proteins are able to form functional homodimers, they predominantly form heterodimers with 14-3-3ζ ([14] and our unpublished observations). Therefore, we tested if *14*-*3*-*3ε* and *14*-*3*-*3ζ* double knockouts show any craniofacial defects resulting from defects in neural crest cell development. We achieved this by producing *14*-*3*-*3ε/14*-*3*-*3ζ* double knockout mice using *Wnt1*-*Cre* transgenic mice in which Cre recombinase is expressed in neural crest cells [15–17].

## Results

**Fig. 1:**
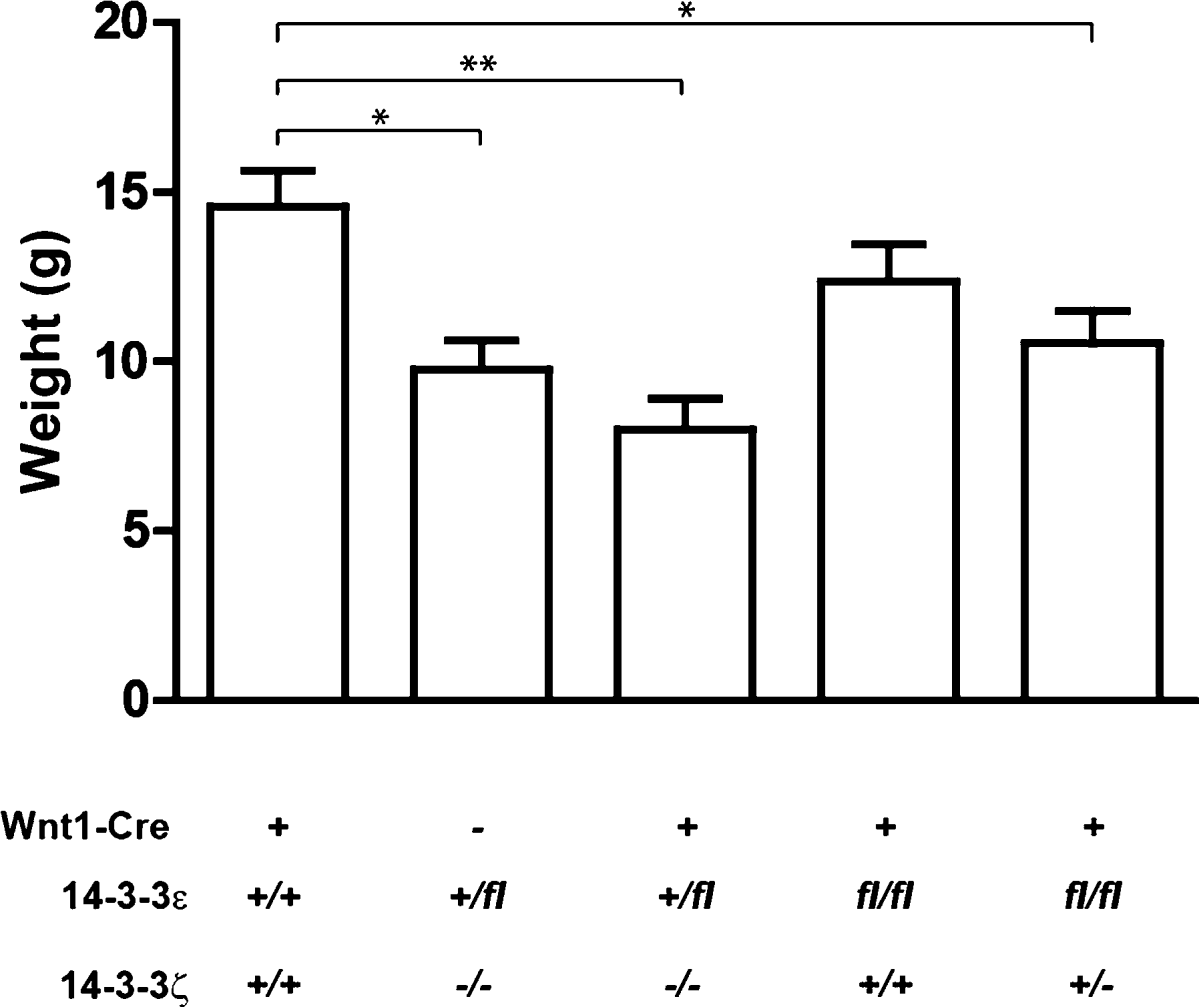
Weight of the *14*-*3*-*3ε/14*-*3*-*3ζ/Wnt1*-*Cre* mice at P21. Weight was measured at P21 and statistical analysis was performed using a one-way ANOVA with the Bonferroni post–hoc test. Values represented as the mean ± SEM. *p < 0.05 and **p < 0.01

To analyze the functions of the 14-3-3ε and 14-3-3ζ proteins in neural crest cells, we utilized mouse genetic approaches using *14*-*3*-*3ε* conditional (flox) knockout mice, *14*-*3*-*3ζ * conventional knockout (KO) mice and *Wnt1*-*Cre* transgenic mice in which Cre recombinase is expressed in the neural crest cells [18]. Although the complete double knockout (*14*-*3*-*3ε*^*fl/fl*^*/14*-*3*-*3ζ*^−*/*−^*/Wnt1*-*Cre*^+^) mice were embryonic lethal, the *14*-*3*-*3ε*^+*/fl*^*/ζ*^−*/*−^*/Cre*^+^ mice are able to survive to adulthood (Table 1). However, the survival rate of the *14*-*3*-*3ε*^+*/fl*^*/ζ*^−*/*−^*/Cre*^+^ mice was lower than expected (Table 1, observed: n = 5, expected: n = 12), and they show decreased weight compared to the control *14*-*3*-*3ε*^+*/*+^*/ζ*^+*/*+^*/Cre*^+^ mice (Fig. 1, Control: 14.58 g ± 2.58, *14*-*3*-*3ε*^+*/fl*^*/ζ*^−*/*−^*/Cre*^+^: 7.98 g ± 2.26).

**Table 1:** Genetic ratio from mating the *14*-*3*-*3ε* ^+*/fl*^ */14*-*3*-*3ζ* ^+*/*−^ */Wnt1*-*Cre* ^+^ mice

Cre	−	−	−	−	−	−	−	−	−	+	+	+	+	+	+	+	+	+
ε	+/+	+/+	+/+	+/fl	+/fl	+/fl	fl/fl	fl/fl	fl/fl	+/+	+/+	+/+	+/fl	+/fl	+/fl	fl/fl	fl/fl	fl/fl
ζ	+/+	+/−	−/−	+/+	+/−	−/−	+/+	+/−	−/−	+/+	+/−	−/−	+/+	+/−	−/−	+/+	+/−	−/−
OBS	8	14	2	15	25	5	8	16	1	6	13	2	14	26	5	5	11	0
EXP	6	12	6	12	24	12	6	12	6	6	12	6	12	24	12	6	12	6

*OBS* observed, *EXP* expected

**Fig. 2:**
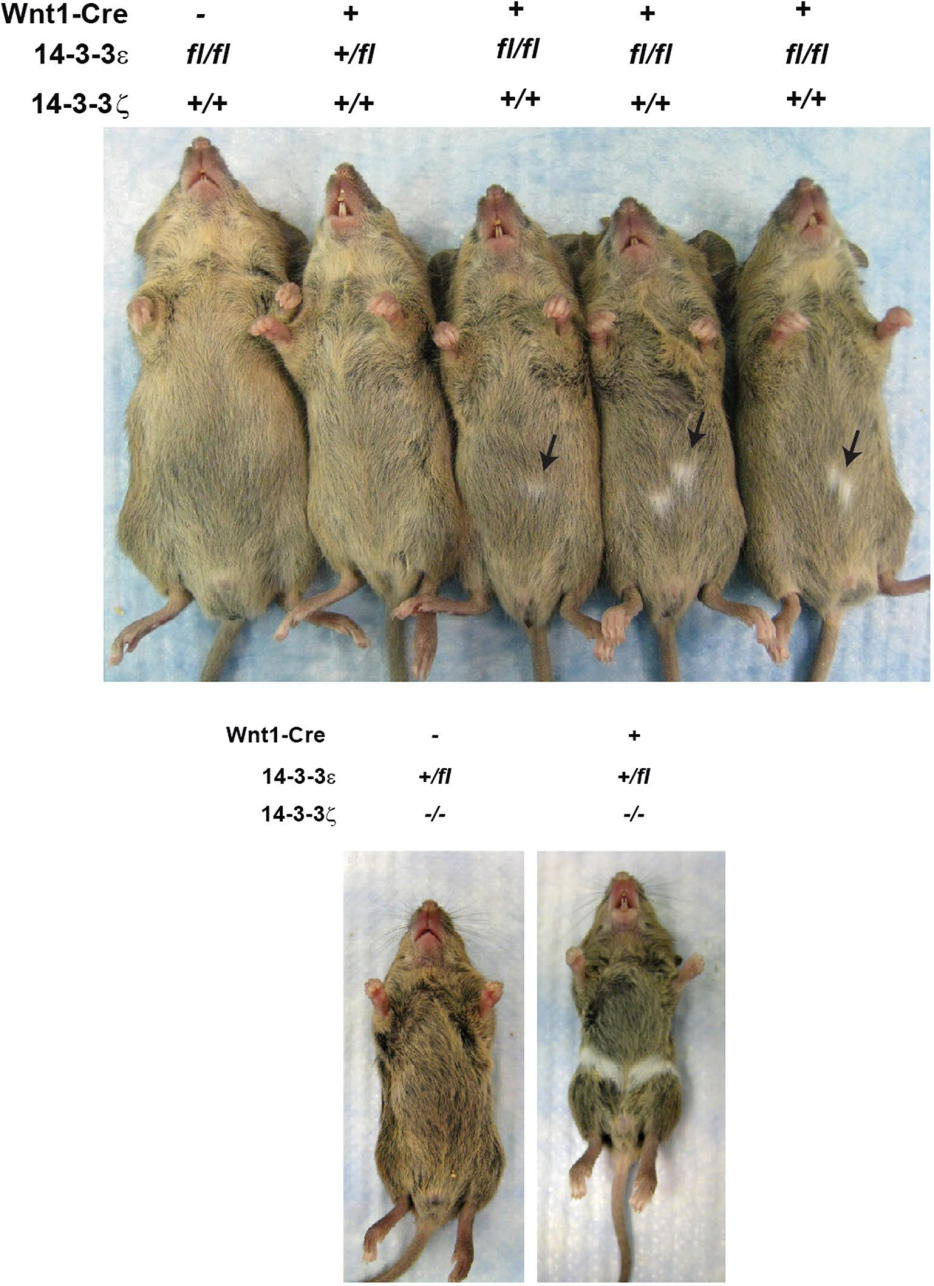
14-3-3 ablation in neural crest cells caused the formation of *white patches* on the ventral region. Photos were obtained at P21. Note that the *14*-*3*-*3ε* ^+*/fl*^ */*ζ^+*/*+^ */Cre* + mice do not have white patches, but the *14*-*3*-*3ε* ^*fl/fl*^ */*ζ^+*/*+^ */Cre* ^+^ mice have *white patches*. *Arrows* in *upper panel* mark *white patches*

The *14*-*3*-*3ε*^+*/fl*^*/ζ*^+*/*−^*/Cre*^+^ mice, *14*-*3*-*3ε*^+*/fl*^*/ζ*^−*/*−^*/Cre*^+^ mice, *14*-*3*-*3ε*^*fl/fl*^*/ζ*^+*/*+^*/Cre*^+^ mice and the *14*-*3*-*3ε*^*fl/fl*^*/ζ*^+*/*−^*/Cre*^+^ mice had white patches of fur on the ventral region of their torso (Fig. 2 and Table 2, *14*-*3*-*3ε*^+*/fl*^*/ζ*^+*/*−^*/Cre*^+^ mice: 88.5 %, *14*-*3*-*3ε*^+*/fl*^*/ζ*^−*/*−^*/Cre*^+^ mice: 80.0 %, *14*-*3*-*3ε*^*fl/fl*^*/ζ*^+*/*+^*/Cre*^+^ mice: 80.0 %, and *14*-*3*-*3ε*^*fl/fl*^*/ζ*^+*/*−^*/Cre*^+^ mice: 100 %). Interestingly, the *14*-*3*-*3ε*^+*/fl*^*/ζ*^+*/*+^*/Cre*^+^ and the *14*-*3*-*3ε*^+*/*+^*/ζ*^−*/*−^*/Cre*^+^ mice did not show this phenotype. However, *14*-*3*-*3ε*^*fl/fl*^*/ζ*^+*/*+^*/Cre*^+^ mice did show white patches on their ventral region. The white patches were observed only on the ventral region of their torso, but not on the tail and paws or any other region. Next, we measured the area of the white patches in each genotype and summarized in Table 3 (*14*-*3*-*3ε*^+*/fl*^*/ζ*^+*/*−^*/Cre*^+^ mice: 0.69 cm^2^, *14*-*3*-*3ε*^+*/fl*^*/ζ*^−*/*−^*/Cre*^+^ mice: 1.09 cm^2^, *14*-*3*-*3ε*^*fl/fl*^*/ζ*^+*/*+^*/Cre*^+^ mice: 0.23 cm^2^, and *14*-*3*-*3ε*^*fl/fl*^*/ζ*^+*/*−^*/Cre*^+^ mice: 0.97 cm^2^). We found that the *14*-*3*-*3ε*^+*/fl*^*/ζ*^+*/*−^*/Cre*^+^ mice, the *14*-*3*-*3ε*^+*/fl*^*/ζ*^−*/*−^*/Cre*^+^ mice, and the *14*-*3*-*3ε*^*fl/fl*^*/ζ*^+*/*−^*/Cre*^+^ mice have larger white patches than the *14*-*3*-*3ε*^*fl/fl*^*/ζ*^+*/*+^*/Cre*^+^ mice. This indicates that neither14-3-3ε or 14-3-3ζ is dominant in regulating the size of the white patches. Together, these data suggest the importance of the 14-3-3 proteins in melanocyte development.

**Table 2:** Observation of mice with white patches

Cre	−	−	−	−	−	−	−	−	−	+	+	+	+	+	+	+	+	+
ε	+/+	+/+	+/+	+/fl	+/fl	+/fl	fl/fl	fl/fl	fl/fl	+/+	+/+	+/+	+/fl	+/fl	+/fl	fl/fl	fl/fl	fl/fl
ζ	+/+	+/−	−/−	+/+	+/−	−/−	+/+	+/−	−/−	+/+	+/−	−/−	+/+	+/−	−/−	+/+	+/−	−/−
OBS	8	14	2	15	25	5	8	16	1	6	13	2	14	26	5	5	11	0
WP	0	0	0	0	0	0	0	0	0	0	0	0	0	23	4	4	11	0
%	0	0	0	0	0	0	0	0	0	0	0	0	0	88.5	80.0	80.0	100	−

*OBS* observed, *WP* number of mice with white patches

**Table 3:** The size of white patches

Cre	+	+	+	+
ε	+/fl	+/fl	fl/fl	fl/fl
ζ	+/−	−/−	+/+	+/−
Average size of white patches (cm^2^)	0.69	1.09	0.23	0.97

**Fig. 3:**
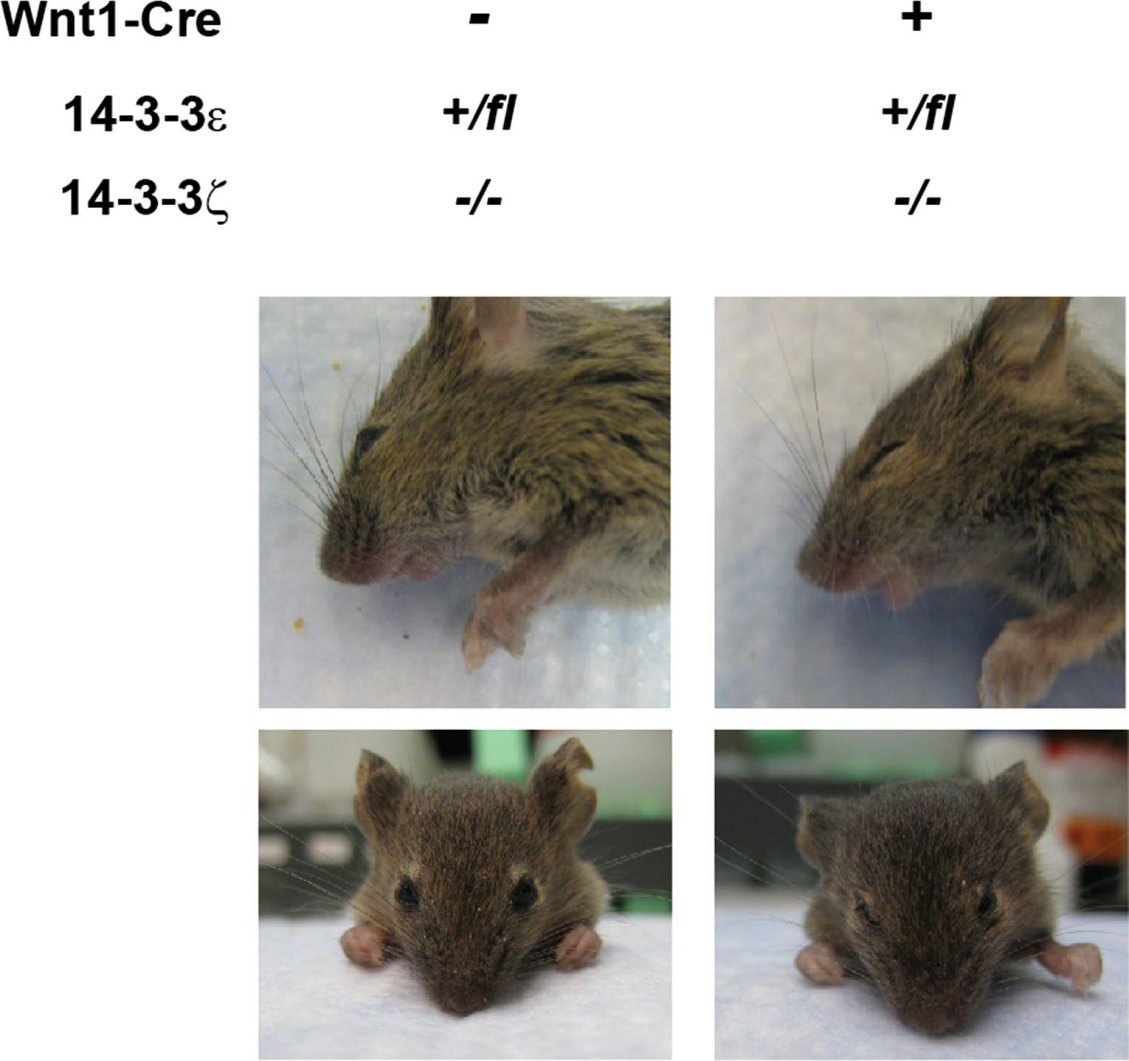
14-3-3 deficiency in neural crest cells did not result in defects in the craniofacial region. Photos were obtained at P21. There were no obvious defects in the craniofacial region in the *14*-*3*-*3ε* ^+*/fl*^ */*ζ^−*/*−^ */Cre* ^+^mice

We also analyzed the craniofacial region for defects since Cre recombinase is also expressed in the craniofacial region (Fig. 3). However, we were not able to find any pronounced defects in the craniofacial region. Further research should be performed to analyze the functions of 14-3-3 proteins in craniofacial development (see the “Discussion” section).

## Discussion

We found that the *14*-*3*-*3ε/14*-*3*-*3ζ/Wnt1*-*Cre* mice had white patches in their fur on the ventral region of their torso. In the *Wnt1*-*Cre* mice, Cre recombinase is expressed in neural crest cells which differentiate into a variety of cells, including melanocytes [18]. A previous study using *Wnt1*-*Cre* mice showed that the AP-2α transcription factor knockout mice had white patches similar to those seen in the *14*-*3*-*3ε/14*-*3*-*3ζ/Wnt1*-*Cre* mice [19]. Neural crest cells are initially generated in the roof plate of the neural tube and migrate and differentiated into specific cells such as melanocytes. Therefore, 14-3-3 proteins could be involved in the migration and differentiation of neural crest cells. Also, it could be possible that 14-3-3 proteins are involved in melanin production in melanocytes. In addition, we cannot exclude the possibility that 14-3-3ζ is important for proper development of neural crest cells because the *14*-*3*-*3ε*^+*/fl*^*/ζ*^+*/*−^*/Cre*^+^ and *14*-*3*-*3ε*^+*/fl*^*/ζ*^−*/*−^*/Cre*^+^ mice showed white patches, but not the *14*-*3*-*3ε*^+*/fl*^*/ζ*^+*/*+^*/Cre*^+^ (Table 2). To avoid the potential functional compensation by other 14-3-3 isoforms during embryonic development, it may be needed to analyze the functions of the 14-3-3ζ protein in pigmentation by creating and analyzing the *14*-*3*-*3ζ* conditional knockout mice in conjunction with the *Wnt1*-*Cre* transgenic mice.

Although there is no statistical significance in the difference in the weight between the *14*-*3*-*3ε*^+*/fl*^*/ζ*^−*/*−^*/Cre*^−^ and *14*-*3*-*3ε*^+*/fl*^*/ζ*^−*/*−^*/Cre*^+^ mice (Fig. 1), the *14*-*3*-*3ε*^+*/fl*^*/ζ*^−*/*−^*/Cre*^+^ mice tend to be smaller than the *14*-*3*-*3ε*^+*/fl*^*/ζ*^−*/*−^*/Cre*^−^ mice (Fig. 1). Obviously, the 14-3-3ζ deficiency results in the smaller body size (Fig. 1). Interestingly, the 14-3-3ε gene was removed by *Wnt1* promoter-driven Cre recombinase although 14-3-3ζ was deleted in all tissues. Therefore, these data suggest that the deletion of the 14-3-3ε protein by *Wnt1* promoter-driven Cre recombinases is responsible for the smaller body size in addition to the 14-3-3ζ deficiency. In addition to neural crest cells, Cre recombinase is expressed in the midbrain/hindbrain junction [16, 20]. Although food consumption was not recorded, it has previously been shown that the hypothalamus is important for controlling feeding behavior [21]. Although the expression of Cre recombinase in the hypothalamus has not been analyzed in *Wnt1*-*Cre* transgenic mice, *Wnt1* mRNA is expressed in the hypothalamus, suggesting the potential expression of Cre recombinase in the hypothalamus in the *Wnt1*-*Cre* mice [22]. Also, a previous study indicated that Cre recombinase is expressed in the pituitary in *Wnt1*-*Cre* mice [23]. It is also known that the functional interaction between the hypothalamus and the pituitary is essential for their functions. Therefore, it is possible that the 14-3-3ε protein is important for the proper function of the hypothalamus and the pituitary and may alter feeding behavior and weight maintenance. To test this hypothesis, the specific ablation of 14-3-3ε in these tissues will need to be analyzed in the future.

In addition to pigmentation defects, it is possible that the ablation of 14-3-3ε and 14-3-3ζ in neural crest cells results in severe defects in other organs and tissues, such as the gastrointestinal tract and thyroid, potentially explaining the lower body weight seen in the *14*-*3*-*3ε/14*-*3*-*3ζ/Wnt1*-*Cre* mice. The enteric nervous system in the gastrointestinal (GI) system is derived from neural crest cells [24, 25]. The enteric nervous system is required for the proper movement of food along the entire GI tract [26]. Disruption of the enteric nervous system therefore could directly impact food uptake and processing and thus interrupt normal growth and weight gain. Therefore, it is possible that the *14*-*3*-*3ε/14*-*3*-*3ζ/Wnt1*-*Cre* mice have defects in GI peristalsis. Also, parafollicular cells, also called C cells in the thyroid, are derived from neural crest cells and secrete calcitonin involved in the regulation of calcium metabolism [27]. Parafollicular cells also secrete other small peptides such as somatostatin and serotonin, and are involved in thyroid hormone production [28, 29]. Therefore, it is possible that the *14*-*3*-*3ε/14*-*3*-*3ζ/Wnt1*-*Cre* mice have defects in controlling hormone production in the hypothalamic-pituitary-thyroid axis due to a dysfunction in these cells or in their localization in the thyroid, which is essential for the regulation of metabolism [28, 29]. Thus, further research should be performed to investigate these potential defects by measuring the concentration of the hormones, such as thyroid stimulating hormone (TSH), T3 and T4, in the *14*-*3*-*3ε/14*-*3*-*3ζ/Wnt1*-*Cre* mice.

Regarding the craniofacial development in the *14*-*3*-*3ε/14*-*3*-*3ζ/Wnt1*-*Cre* mice, we were not able to find any significant defects in the craniofacial region. However, more research on this topic needs to be undertaken before reaching a conclusion. Further experiments including histology and immunohistochemistry such as bone staining by Alcian Blue/Alizarin Red should be done. Also, it is very helpful for better understanding the mechanisms of craniofacial development to use other *Cre* transgenic mice, including earlier developmental *Cre* expression than the *Wnt1* promoter can provide, and compare the results obtained from the analyses using these different *Cre* transgenic mice. The *P0 (protein 0)*-*Cre* transgenic mouse is another frequently used mouse line in which *Cre* recombinase is expressed in epithelial layers of developing tooth germ and taste buds [30]. Also, another *Cre* transgenic line, tamoxifen-inducible *Sox10*-*Cre* transgenic mice in which the expression of Cre recombinases can be regulated by the administration of tamoxifen, will be useful to analyze potential defects in greater detail [31, 32]. Thus, a combinatorial use of a different Cre transgenic mouse lines will provide knowledge for understanding the precise mechanisms of craniofacial development.

## Conclusions

Analysis of the functions of 14-3-3ε and 14-3-3ζ proteins indicates their importance in the development of neural crest cells, in particular the development of the melanocyte lineage. Also, our data suggest that the 14-3-3ε proteins are important for weight modulation during development.

## Methods

### Mice

The *14*-*3*-*3ε* conditional (flox) mice and the *14*-*3*-*3ζ* conventional knockout (KO) mice are described previously [2, 4]. The *14*-*3*-*3ε* flox mice and the *14*-*3*-*3ζ* KO mice have been maintained in the 129 genetic background by continuing to backcross them with 129SVE inbred strain (Taconic Biosciences, Inc.) for more than twenty generations. The *Wnt1*-*Cre* transgenic mice were obtained from the Jackson Laboratory (STOCK Tg (Wnt1-cre) 11Rth Tg (Wnt1-GAL4)11Rth/MileJ, C57BL/6 genetic background) and have been maintained by crossing with 129SVE. Therefore, all mice used in this study were congenic strain with 129SVE genetic background. Genotyping was performed using tail clippings and PCR with specific primers as previously described [2, 4, 18]. All experiments were performed following protocols approved by the Drexel University Animal Care and Use Committees.

### Statistical analysis

Statistical analysis of mouse weight was performed using Prism (GraphPad Software). The data were analyzed by one-way ANOVA with the Bonferroni post hoc test. Results were deemed statistically significant if the *p* value was <0.05. *p < 0.05 and **p < 0.01.

## Abbreviation

ANOVA: analysis of variance
